# High positive predictive value of CNVs detected by clinical exome sequencing in suspected genetic diseases

**DOI:** 10.1186/s12967-024-05468-1

**Published:** 2024-07-09

**Authors:** Yimo Zeng, Hongke Ding, Xingwang Wang, Yanlin Huang, Ling Liu, Li Du, Jian Lu, Jing Wu, Yukun Zeng, Mingqin Mai, Juan Zhu, Lihua Yu, Wei He, Fangfang Guo, Haishan Peng, Cuize Yao, Yiming Qi, Yuan Liu, Fake Li, Jiexia Yang, Rong Hu, Jie Liang, Jicheng Wang, Wei Wang, Yan Zhang, Aihua Yin

**Affiliations:** 1 Medical Genetics Center, Guangdong Women and Children Hospital, Xingnan Road 521, Guangzhou, 510010 Guangdong China; 2 Maternal and Children Metabolic-Genetic Key Laboratory, Guangdong Women and Children Hospital, Guangzhou, China; 3 Guangzhou Key Laboratory of Prenatal Screening and Prenatal Diagnosis, Guangzhou, China

**Keywords:** Copy number variations, Chromosome microarray, Exome sequencing, Multiplex ligation-dependent probe amplification assay, Real-time quantitative polymerase chain reaction

## Abstract

**Background:**

Genetic disorders often manifest as abnormal fetal or childhood development. Copy number variations (CNVs) represent a significant genetic mechanism underlying such disorders. Despite their importance, the effectiveness of clinical exome sequencing (CES) in detecting CNVs, particularly small ones, remains incompletely understood. We aimed to evaluate the detection of both large and small CNVs using CES in a substantial clinical cohort, including parent–offspring trios and proband only analysis.

**Methods:**

We conducted a retrospective analysis of CES data from 2428 families, collected from 2018 to 2021. Detected CNV were categorized as large or small, and various validation techniques including chromosome microarray (CMA), Multiplex ligation-dependent probe amplification assay (MLPA), and/or PCR-based methods, were employed for cross-validation.

**Results:**

Our CNV discovery pipeline identified 171 CNV events in 154 cases, resulting in an overall detection rate of 6.3%. Validation was performed on 113 CNVs from 103 cases to assess CES reliability. The overall concordance rate between CES and other validation methods was 88.49% (100/113). Specifically, CES demonstrated complete consistency in detecting large CNV. However, for small CNVs, consistency rates were 81.08% (30/37) for deletions and 73.91% (17/23) for duplications.

**Conclusion:**

CES demonstrated high sensitivity and reliability in CNV detection. It emerges as an economical and dependable option for the clinical CNV detection in cases of developmental abnormalities, especially fetal structural abnormalities.

**Supplementary Information:**

The online version contains supplementary material available at 10.1186/s12967-024-05468-1.

## Introduction

Intellectual disability, schizophrenia, cerebral palsy and various other genetic disorders are significantly influenced by CNVs [1–5]. Numerous studies have highlighted the pivotal role of CNVs in various genetic disorders, particularly fetal structural anomalies, and pediatric neurological conditions [6, 7]. Evidence suggests that roughly 6% of clinically significant chromosomal irregularities manifest in fetuses with structural anomalies, even those with a normal karyotype [6]. Whether occurring independently or in conjunction with Single Nucleotide Variations (SNVs), CNVs, particularly smaller ones, contribute significantly to the genetic landscape of disorders [8]. However, the lack of an efficient method for simultaneous CNV and SNV detection poses a significant challenge in genetic diagnostics. While Exome Sequencing (ES) has emerged as a secondary option following negative CMA results, its sequential approach contradicts the clinical mandate for timely and cost-effective solutions.

In recent years, exome sequencing has provided valuable insights into CNVs spanning coding regions and detecting CNVs smaller than 1000 bp [9, 10], positioning ES as a promising alternative for CNV detection [11, 12]. Researchers increasingly favor WES for CNV detection [12–16], recognizing the advantages of analyzing raw data from next-generation sequencing (NGS) [12]. In 2022, Testard et al. concluded that WES could serve as a primary test for CNV detection after analyzing 2418 cases [17]. However, this study employed various ES scenarios and lacked systematic validation of each CNV identified. Despite the potential of WES in CNV identification, many reports on CNV detection rates in ES lack accuracy assessments due to variations in CNV calling tools and algorithms used.

Despite CMA being an initial CNV detection method, its limitations in detecting small CNVs and low mosaic levels are apparent. Growing evidence supports WES's potential in CNV identification. Leveraging WES's simultaneous detection of CNVs and SNVs could revolutionize genetic disorder diagnosis, substantially reducing costs and turnaround times. Thus, our study aims to rigorously evaluate clinically relevant CNV detection in CES through diverse validation methods, ensuring its reliability and clinical utility.

## Methods

### Subjects

6465 individuals in 2428 unrelated pedigrees were recruited at the Medical Genetic Center in Guangdong Women and Children Hospital between June 2018 and November 2021. Of these, 2328 families underwent genetic testing for etiology diagnosis while an additional 100 families sought carrier screening due to a history of undiagnosed abnormal pregnancy. CES analysis was performed on 2375 fetuses or probands, including 1894 fetus-parental trios, 47 sets of twins, and 8 fetus-parent dyads. Various sample types were collected, and chromosomal abnormalities were excluded though thorough examination. Peripheral blood samples were collected from 1006 families, one tissue sample was taken from a deceased newborn, while the remaining 1421 families provided fetal samples (325 chorionic villus, 484 amniotic fluid, 496 umbilical cord blood, 115 fetal tissue and one fetal heart blood sample). Due to the long turnaround time of CES, for fetuses with serious ultrasound abnormalities found in third trimester, such as hydrocephalus and fetal edema, the cases were not included in the cohort if the pregnant woman decided to terminate the pregnancy. This study adhered to the principles of the Declaration of Helsinki and received approval from the Institutional Review Board of Guangdong Women and Children Hospital. All subjects provided informed consent, with parental constant obtained for participants under eight years old or fetuses.

### CES and CNV calling

The DNA was extracted from various original samples including peripheral blood, villi, amniotic fluid, and tissues. After the DNA library developing with the standard procedure, exome targets were captured with a custom-designed Medical Exome capture kit (AmCare Genomic Lab, Guangzhou, China). This kit specifically covers the coding region of about 4000 morbid genes corresponding to human genetic diseases in OMIM database (Supplement 1). Subsequently, the captured libraries were then sequenced using the Illumina HiSeq platform (Illumina, Inc., San Diego, CA, USA) to generate raw paired-end reads of 150 base pairs each. Quality control measures were applied to filter out adapter sequences and low-quality reads before mapping them to the reference genome through fastp with default parameters [18]. BWA was employed to align high-quality reads to the Homo sapiens reference genome (hg19) [19]. Picard tools were then used to sort and mark PCR duplicate reads, generating BAM files. Variant calling was performed using the GATK HaplotypeCaller [20]. CNV calling was performed using ClinCNV which is based on an original algorithm that combines the circular binary segmentation method and Hidden Markov model-based approaches [21]. The software utilizes coverage depth as an indicator of CNVs by comparing read counts between test samples and a control cohort under the assumption that read count is proportional to genetic material quantity within a specific region. CES data sets of 200 individuals without clinical phenotypes served as the control cohort. CNVs were filtered for log-likelihood ≥ 20.00 (scaled by regions) and q-value ≤ 0.05. The filter CNVs were further annotated by AnnotSV [22]. After normalizing the bin signals, each small CNV with an AnnotSV pathogenic prediction score higher than 3 was inspected manually to eliminate obvious false positive events from batch effect. Standards and guidelines recommended by the American College of Medical Genetics and Genomics and the Association for Molecular Pathology were used to interpret the CNV variants [23].

### CNVs validation

All kinds of CNVs found by CES need to be validated. When multiple samples contain the same CNVs, one or several of them were randomly selected for verification (a CNV was not validated due to an insufficient sample). When validating CNVs, sample selection followed the following criteria: all samples exhibiting a CNV detected only once underwent validation; for samples with CNVs occurring between 1 and 10 times, 25% were randomly selected or at least one sample was chosen for validation; for samples with CNVs occurring more than 10 times, 15% were randomly selected for validation.

Various techniques were employed to validate the CES-detected CNVs, including CMA, CNV-Seq, and PCR-based methods such as MLPA, custom-designed liquid phase chip analysis, qPCR, Gap-PCR, and Sanger sequencing. The specific operational procedures for each validation technique were listed in the Supplement 2. For CNVs larger than 100 kb (deletion) or 500 kb (duplication), validation was performed using CMA (Affymetrix 750 k) or CNV-seq (Proton Sequencer from Thermo Fisher Scientific). PCR-based techniques were utilized for validating CNVs smaller than 100 kb (deletion) or 500 kb (duplication), with priority given to commercial reagent kits followed by specially designed PCR assays. Frequent CNVs like 16p11.2 and 16p13.3 were verified using MLPA kits (SLASA; MLPA Probemix P102-D1 HBB; P140-C1 HBA; P034/035-B1 DMD; and P055-C1 PAH from MRC-Holland) and custom-designed liquid phase chip analysis (Supplement 3). Uncommon CNVs were confirmed through Gap-PCR and qPCR assays (Supplement 3).

During the process of verifying CNVs using different tools, distinct criteria were applied to determine consistency. For CMA, CNV-seq, MLPA, and custom-designed liquid phase chip analysis, if more than half of the length of a verified method’s detected CNV coincided with that detected by CES it was considered consistent [24]; otherwise, it was deemed inconsistent. For Gap-PCR and Sanger sequencing—obtaining an amplified fragment with the expected length or identifying a breakpoint constituted consistency. Otherwise, it would be considered inconsistent. The qPCR primers were designed according to the range of CES detection indicating, and the *RPP30* gene was used as an internal reference (primers were available in Supplement 3). One pair of specific primers was designed for each target CNV. Copy number results obtained from qPCR were determined based on the MLPA reagent standard: Relative quantification (RQ) ranging from 0.40 to 0.65 indicated one copy; 0.80 to 1.20 represented two copies, while > 1.30 indicated three copies or more. If the results suggest the same type of CNV. We determined that it was consistent with CES.

If the verification results were consistent with CES, other techniques would not be considered; if not, additional validation would have needed to be done with other methods. A few CNVs smaller than 100 kb (deletion) or 500 kb (duplication) were confirmed by CMA and/or CNV-seq due to difficulties in implementing qPCR (Cases 23, 24, 25, 48, 91 and 93).

### Statistical analysis

The enumeration and classification of copy number variations are described utilizing frequency or composition ratios. The positive predictive value is employed to characterize the efficacy of the detection methodology. The 95% confidence interval of the positive predictive value is computed via the exact binomial test. Statistical analyses were performed using R version 4.0.2.

## Results

### CNV detected by CES and its distribution

**Fig. 1:**
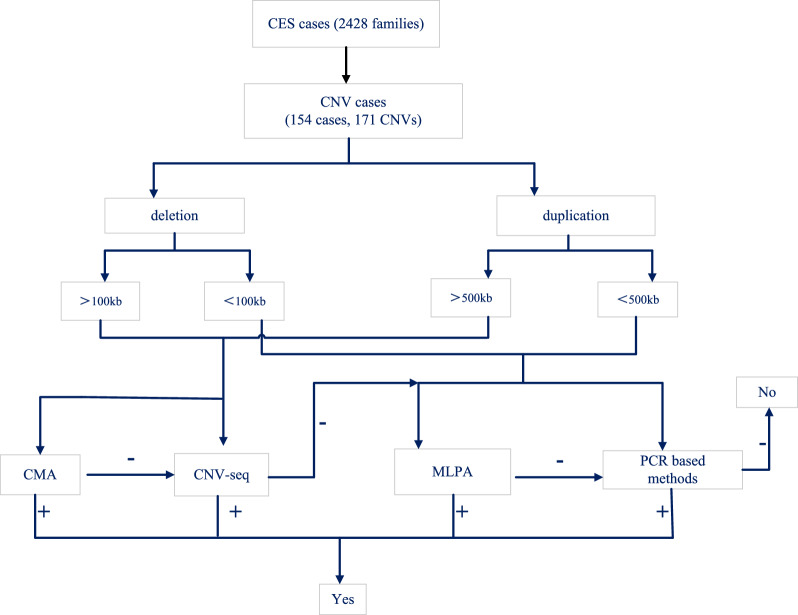
Flowchart of the study. 171 CNVs in 154 cases were detected in 2428 unrelated families by CES. Four different validation methods were employed to confirm these CNVs. Notably, CMA and CNV-seq demonstrated a higher preference for confirming CNVs larger than 100 kb (deletion) or 500 kb (duplication), while MLPA and PCR-based methods were utilized for CNVs smaller than 100 kb (deletion) or 500 kb (duplication). In cases of consistent validation results, the respective CNV was considered as true positive; however, if inconsistencies arose, an alternative validation method was employed

**Fig. 2:**
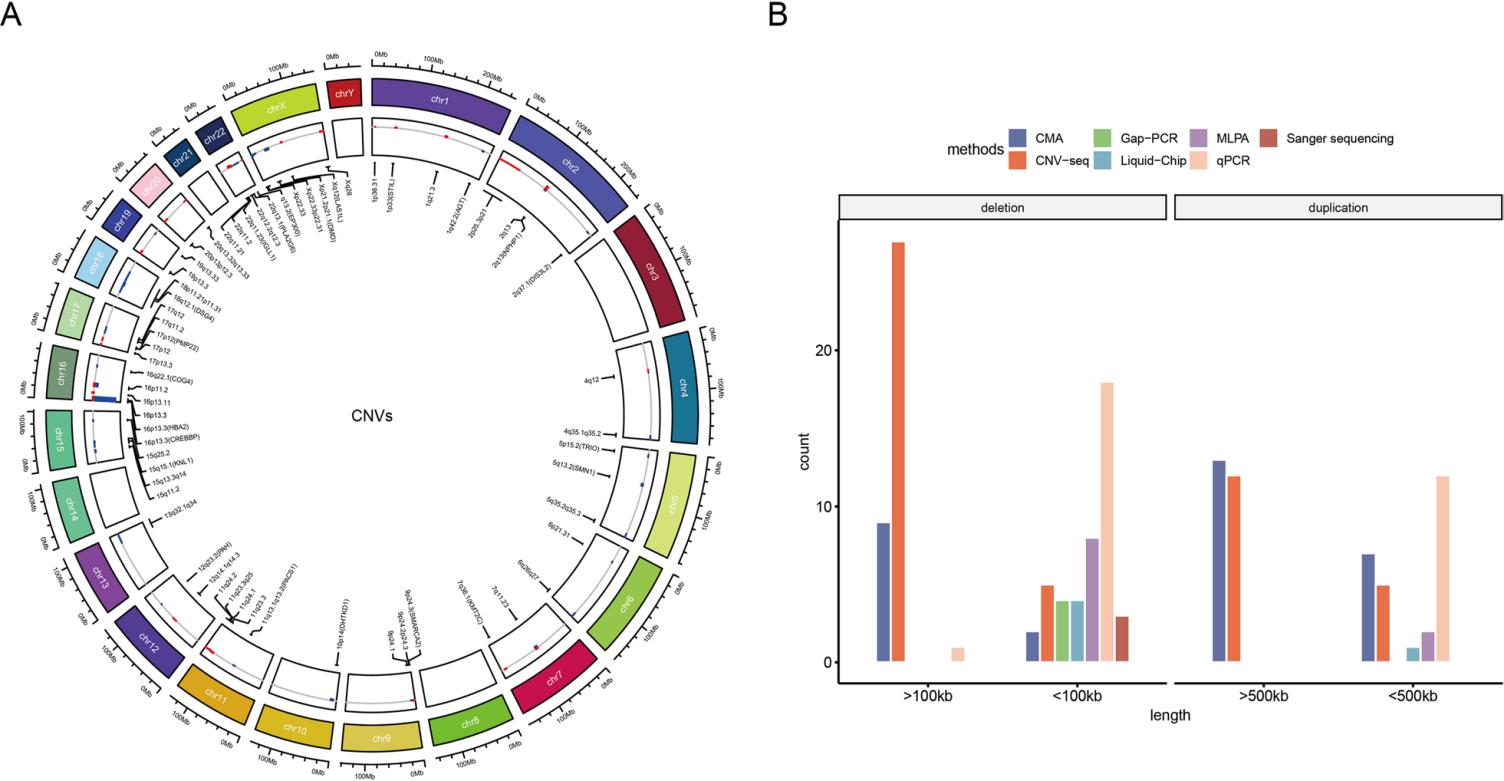
Distribution of CNVs in the genome and validation methods. **A** The tracks, from outer to inner circles, include: (1) ideogram-based representation of chromosomes; (2) CNVs, with red indicating duplications, blue indicating deletions, and the height of bars reflects the number of CNVs; and (3) chromosomal regions corresponding to CNVs. **B** Different validation methods were used for various groups of CNV. CMA and CNV-seq were mainly used for large fragments, while multiple methods were employed for small fragments

The CES identified a total of 171 clinically relevant CNVs, which were further classified into deletions and duplications. These CNVs were then sub-grouped based on size (> 100 kb and ≤ 100 kb; > 500 kb and ≤ 500 kb), resulting in 117 deletions (32 CNVs > 100 kb, 85 CNVs ≤ 100 kb) and 54 duplications (23 CNVs > 500 kb, 31 CNVs ≤ 500 kb) (Fig. 1, Supplement 4). These CNVs were distributed throughout the genome except for chromosome 3, 8, 14, 21, and Y (Fig. 2A). Among the CNVs, 16p accounted for 40.69% (70 CNVs) of the total, which included 54 CNVs in 16p13.3, five CNVs in 16p13.11, and 11 CNVs in 16p11.2 (Supplement 4). In addition to these prominent hotspots, there were also 31 recurrent CNV regions and 32 rare CNV regions identified.

### CNVs validation

Following selection of representative cases for each type of CNV, a total of 103 cases (113 CNVs) (Supplement 5) were validated.

In total, there were 31 CNVs validated by CMA (9 deletions > 100 kb, 2 deletions ≤ 100 kb, 13 duplications > 500 kb, and 7 duplications ≤ 500 kb), 49 CNVs validated by CNV-seq (27 deletions > 100 kb, 5 deletions ≤ 100 kb, 12 duplications > 500 kb, and 5 duplications ≤ 500 kb), 10 CNVs validated by MLPA (8 deletions ≤ 100 kb, and 2 duplications ≤ 500 kb) and 40 CNVs were confirmed by PCR based methods (1 deletion > 100 kb, 26 deletions ≤ 100 kb, and 13 duplications ≤ 500 kb) (Fig. 2B). Among all the validated CNVs, 94 were confirmed using a single method, 18 using two methods, and 1 using three methods.

CNVs larger than 100 kb (deletion) or 500 kb (duplication) found by CES were all consistent with CMA or CNV-Seq, resulting in PPVs of 100%. Among small CNVs, 30 out of 37 deletions (81.08%) and 17 out of 23 duplications (73.91%) were in accordance with validated methods (Table 1).

**Table 1:** Validation results of CNVs

Subgroups		CNV No. found by CES	Confirmed CNV No. using other methods	Positive predictive value (PPV)
Deletion	> 100 kb	31	31	100% (88.4–100%)
	≤ 100 kb	37	30^a^	81.08% (64.84–92.04%)
Duplication	> 500 kb	22	22	100% (84.6–100%)
	≤ 500 kb	23	17	73.91% (51.59–89.77%)
Total		113	100	88.49% (81.13–93.73%)

^a^Case 46 should be firmly classified as non-compliant based on the judgment criteria, as detailed in the results section

There were 13 small CNVs in 13 instances (cases 31, 36, 39, 40, 47, 56, 57, 84, 85, 87, 90, 95, and 102), which did not match with validated methods. These CNVs were deletions ranging from 0.4 kb to 81 kb encompassing multiple exons of a single gene (e.g., exon 24–25 of *KNL1* (NM_170589) and exon 21–22 of *CREBBP* (NM_004380)) and duplications ranging from 4.4 kb to 126 kb involving several exons of one gene (e.g., exon 1–6 of *EP300* (NM_001429), exon 14–16 of *STIL* (NM_001282939), and exon 9–59 of *KMT2C* (NM_170606)) or more than one gene (11q23.3).

We conducted thorough follow-up on CES-CNV-affected cases whose results could not be validated. In case 36, a compound heterozygous deletion of exon 13–17 in the maternal allele combined with a paternal c.1547C > T pathogenic variation in *PLA2G6* (NM_003560) was identified. The proband exhibited gait problems, developmental regression (DQ 46.6), and an abnormal occipital cistern via head MRI at the age of 4, consistent with *PLA2G6*-linked infantile neuroaxonal dystrophy 1 (OMIM#256600), suggesting the unidentified CNV could be genuine. Cases 56 and 57, twins, had a likely pathogenic deletion in *KNL1* (NM_170589) exon 24–25 compounded with paternal c.4914_4915 duplication. Additionally, case 56 exhibited a further deletion of 398 kb in region 16p11.2. Unfortunately, one of the twins (case 57) died shortly after birth due to low birth weight; however, the surviving infant was followed up until reaching ages of 3 years and 7 months, showing normal language and mental development without any clinically relevant phenotype. There was no obvious phenotype during follow-up in two patients (cases 84 and 85) with 11q23.3 duplications. *KMT2C* (NM_170606) duplication was found in two cases (cases 90 and 95), which were also present in their mother, however, none of them exhibited any phenotype during follow-up. An entire *NPHP1* (NM_000272) heterozygous deletion was identified in two cases (cases 39 and 40), but no manifestations were observed. In case 102, a de novo duplication spanning exon 1–6 of *EP300* (NM_001429) was discovered along with a deletion of 22q12.2q12.3 (> 3.07 Mb). The patient’s phenotype was consistent with the characteristics of the 22q12.2 deletion syndrome; however, no corresponding phenotype related to *EP300* was detected. Case 31 presented with a complete heterozygous deletion of *IGLL1* (NM_020070), while case 88 had a duplication of 16p13.3 (> 4.4 kb), neither exhibiting a distinct clinical manifestation associated with these genetic abnormalities. Case 47 had a de novo deletion of *CREBBP* (NM_004380) exon 21–22, the fetus was terminated during the pregnancy due to unilateral limb absence and talipes equinovarus deformity. The exact clinical phenotype related to the *CREBBP* gene abnormality could not be determined based on available evidence. According to the current technologies and clinical data, the authenticity of CNV carried by these inconsistent cases cannot be accurately judged, and further studies are needed.

**Fig. 3:**
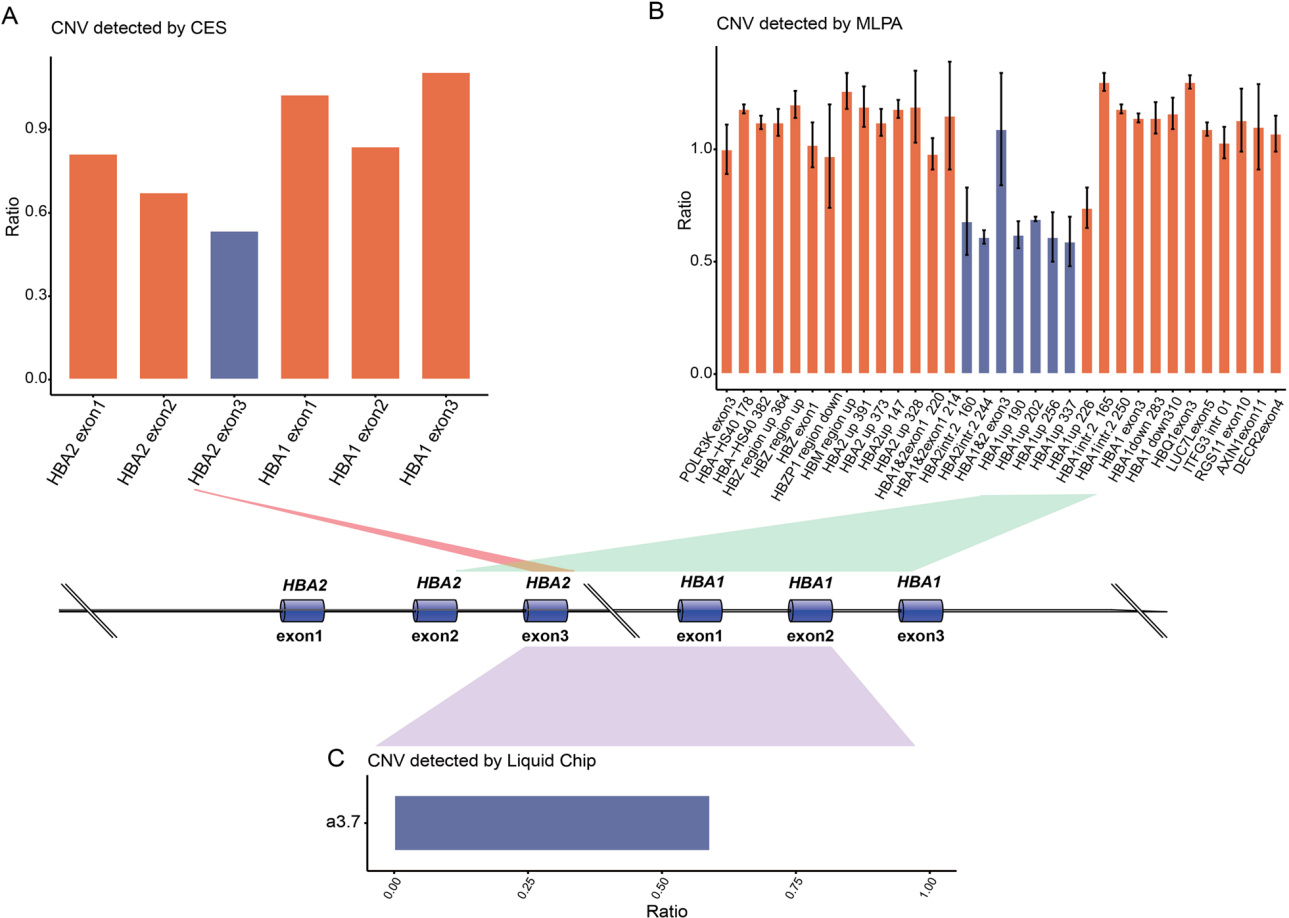
Verification results of case 46. **A** CES indicated a deletion of exon 3 of *HBA2*. **B** MLPA indicated a deletion in *HBA1* (NM_000558.5). **C** Schematic diagram of liquid phase chip for thalassemia detection; the purple represents the deletion

Apart from these inconsistent cases mentioned above, we encountered an intriguing case (case 46) in which CES indicated a deletion of exon 3 of *HBA2* (NM_000517) (Fig. 3A). Subsequent validation using MLPA as the primary method revealed a larger extent of deletion (Fig. 3B). According to consistency criteria, this result did not match with the CES finding. A custom-designed liquid phase chip for gene detection associated with thalassemia was then used and confirmed partial deletions of *HBA2* (NM_000517) and upstream *HBA1* (NM_000558.5) (-α3.7) (Fig. 3C). Due to the presence of homologous sequence, MLPA lacks precision in identifying *HBA1/2* genes and accurately detecting decreased signal from exon 3 when only one out of four copies is deleted.

**Fig. 4:**
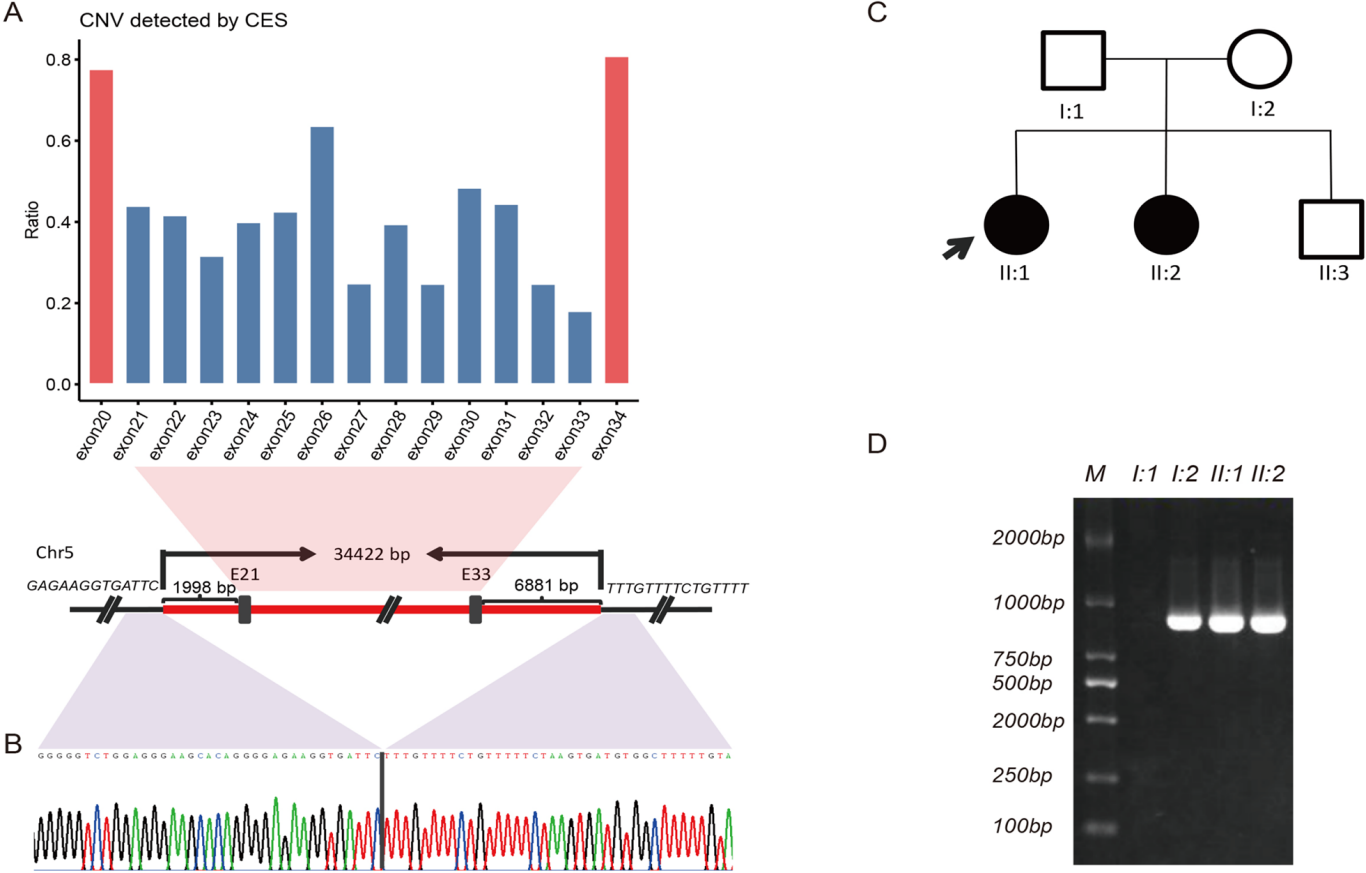
Verification results of case 103. **A** CES indicated a deletion of exons 21 to 33 of *TRIO* (NM_007118). **B** Combining gap-PCR with Sanger sequencing detected the breakpoints in *TRIO* (NM_007118). **C** Family diagram of case 103. **D** PCR validation of CNV in the family using primers designed to flank the breakpoint

Case 103 involved a family in which two girls had intellectual disabilities. CES indicated a de novo deletion of exon 21–33 in the *TRIO* gene (NM_007118) in the proband (II-1). The breakpoint was subsequently identified by Gap-PCR and Sanger sequencing (34,422 bp deletion). The CNV was detected in both the proband's younger sister and their mother with Gap-PCR (Fig. 4). After rechecking the NGS data, a mosaic deletion was found in the mother.

## Discussion

In this study, we found 171 clinically significant CNVs in 154 unrelated cases from a total of 2428 pedigrees. These CNVs were detected through a capture-based CES approach, utilizing the Genome Analysis Toolkit (GATK) algorithm and ClinCNV. The prevalence of clinically relevant CNVs was determined to be 6.3% (154/2428), aligning with findings from previous studies employing WES or CMA [25]. Expectedly, we observed CNV hotspots such as 16p13.3, 16p11.2, and 22q11.21 [26–28]. Moreover, these CNVs were distributed throughout the genome, with few chromosomes showing no detectable alterations, indicating robust clinical representation within our cohort. The absence of CNVs on chromosomes 3, 8, 14, 21, and Y in our study could be attributed to their low incidence rate and the association with intellectual disabilities [29–33]. However, due to limited cases of intellectual disabilities in our cohort, further exploration of these associations was challenging. In addition, certain CNVs, such as the *AZF* deletion on the Y-chromosome were excluded from our study since they are primarily associated with infertility.

The overall concordance between CES and other CNV detection methods was 88.49%. With the inclusion of recurrent low penetrance CNVs or recessive CNV carrier events [16p13.3 deletion (n = 50), 16p11.2 deletion (n = 9), 1q21.3 duplication (n = 3), *SMN1* deletion (n = 4), *DMD* deletion (n = 4), *NPHP1* duplication (n = 8)], the reliability of CNV calls made by CES increased to 92.39%. This concordance rate of our CNV detection is comparable to the exon-resolution CMA detection rate (89%) reported by Petr Danecek et al. in multicenter studies [34]. Furthermore, our study benefited from a larger sample size, minimizing bias compared to earlier similar studies with smaller cohorts [14, 15]. Notably, we employed a range of verification techniques to validate detected CNVs, enhancing the reliability of CES-based CNV identification. Thus, this study represented the first systematic evaluation of capture-based ES-derived CNVs reliability.

All 53 CNVs larger than 100 kb (deletion) or 500 kb (duplication) were confirmed by CMA or CNV-seq, affirming the credibility of larger CNVs detected by CES (Table 1). Additionally, 78.33% (47/60) of small CNVs were confirmed by other methods, while the remaining 21.67% (13/60) were excluded from the simultaneous validation due to their location in highly homologous genomic regions, e.g., exons 2–3 of *IGLL1* (NM_020070) gene, increasing the likelihood of alignment errors in short-read sequencing data.

Our study underscores the authenticity of CNVs identified through CES, particularly for larger CNVs, which are significant to genetic lab scientists and clinicians. To ensure optimal efficacy, a combination of validation strategies should be employed in clinical settings, considering factors such as cost, turnaround time and CNV size and location. CMA or CNV-seq are recommended for validating larger CNVs, while smaller CNVs can be verified using flexible approaches based on available resources and CNV characteristics.

However, the detection of CNVs using CES is subject to limitations. Firstly, the analysis is confined to coding regions associated with Mendelian diseases. Moreover, the presence of genomic regions with homologous sequences introduces bias in read mapping and CNV calling. While CMA remains the gold standard for validating large CNVs, various methods including MLPA, PCR-based approaches, custom-designed liquid phase chip, and qPCR can be utilized for validating small CNVs [35, 36].

For instance, in the case of alpha thalassemia, the highly homologous sequences between *HBA1* (NM_000558.5) and *HBA2* (NM_000517), pose challenges in accurate identification. The common 3.7 kb deletion leads to fusion of exon 1 and 2 from *HBA2* (NM_000517) with exon 3 from *HBA1* (NM_000558.5). This deletion eliminates one instance of exon 3, one of the four copies exhibiting a consensus sequence within the *HBA1*/*2* gene locus. This phenomenon likely explains discrepancies between CES suggesting a deletion encompassing exon 3 in *HBA2* and inconclusive MLPA findings, although case 46 hinted at a substantial deletion. Additionally, the custom-designed liquid phase chip for thalassemia gene detection suggested a genotype of -α3.7/αα. Despite inconsistencies between MLPA results and those from the custom-designed liquid-phase chip, CES consistently indicated the presence of the deletion, providing valuable insights cross-validated using alternative methods. Consequently, owing to its informative indication, the deletion was categorized within the cohesive subgroup, as delineated in Table 1.

This approach is not only cost-effective and time-saving, but also meets the clinical requirements of "accurate and timely" diagnosis, providing benchmarks for establishing clinical pathways. For other small CNVs, the verification scheme can be flexibly selected based on CNV size, genomic location.

Several limitations of our study should be acknowledged, including retrospective data from a single center, lack of long-term follow-up data, especially for unconfirmed CNVs, and insufficient case studies on chimerism. More extensive multi-center research and additional cases are needed to further refine our verification procedure.

## Conclusions

CES accurately provides CNV information for genetic disease cases, offering distinct advantages over CMA, especially in detecting small CNVs. Thus, it serves as a highly effective method for CNV detection in clinical practice.

### Supplementary Information

Supplementary Material 1.

Supplementary Material 2.

Supplementary Material 3.

Supplementary Material 4.

Supplementary Material 5.

## Data Availability

All data generated and/or analyzed during this study are included in this article and its Supplemental information files.

## Abbreviations

CNVs: Copy number variations
ES: Exome sequencing
CES: Clinical exome sequencing
CMA: Chromosomal microarray analysis
MLPA: Multiplex ligation-dependent probe amplification assay
WES: Whole exome sequencing
NGS: Next-generation sequencing
