# Supplementary material for: High positive predictive value of CNVs detected by clinical exome sequencing in suspected genetic diseases
Source: J Transl Med. 2024 Jul 9;22:644. doi: 10.1186/s12967-024-05468-1 (PMC11234535; doi:10.1186/s12967-024-05468-1)
Supplement: Supplementary file 2 — Supplementary Material 2. [file 12967_2024_5468_MOESM2_ESM.docx]

**The procedure of validation methods**

**1. CMA**

Genomic DNA was extracted using a DNA extraction kit (QIAamp DNA Mini Kit, QIAGEN, Germany). Chromosomal microarray analysis (CMA) was performed using a whole-genome CytoScan 750K array (Thermo Fisher Scientific, USA), following the manufacturer’s recommendations. In accordance with standardized procedures, 250 ng of DNA was digested, ligated, amplified, purified, fragmented, labeled, and hybridized probes. The hybridized probes were washed in phosphate buffer and detected using a laser scanner. The raw data were analyzed using Chromosome Analysis Suite 4.0 (Thermo Fisher Scientific, USA) based on the GRCh37/hg19 genome version.

**2. CNV-seq**

CNV-seq was conducted following the methodology outlined in our previous study with minor modifications [1]. In summary, genomic DNA ranging from 100 to 350 ng was fragmented, and DNA libraries were prepared through adapter ligation and PCR amplification (following the instructions provided with the NEB Next dsDNA Fragmentase Kit). Subsequently, the DNA library underwent sequencing using an Ion Proton Sequencer (Thermo Fisher Scientific, Waltham, MA, USA), generating approximately 4 to 5 million raw single-ended sequenced reads with an average length of around 200 bp. Utilizing the Burrows-Wheeler algorithm, 2.5-3.5 million uniquely mapped reads were aligned with the GRCh37/hg19 genome version and allocated to a 20 kb bin on each chromosome. To mitigate the impact of GC bias across different samples, a sequence of three processes, including loess regression, temporal normalization and linear model regression, were employed. Finally, the cyclic binary segmentation (CBS) algorithm was applied to identify CNVs.

3. **Multiplex ligation‑dependent probe amplification (MLPA)**

MLPA was conducted using SALSA MLPA Probe-mix P102-D1 HBB, P140-C1 HBA, P034/035-B1 DMD, P055-C1 PAH (MRC-Holland, Amsterdam, the Netherlands) following the manufacturer’s protocols. In brief, DNA was diluted to 30 ng/µL, and 5 µL of DNA was added to each test tube and placed in a thermocycler. The program was initiated at 98°C for 5 min, followed by cooling at 25°C. Then, 1.5 µL of the prepared probe mixture and 1.5 µL of MLPA buffer were added and incubated at 95℃ for 1 min, followed by 60℃ for 16 h. Subsequently, the thermocycler temperature was reduced to 54℃, and 32μL ligase mixture was added into each tube, along with 1μL of Ligase-65. After incubating at 54℃ for 15 min, followed by 98℃ for 5 min the mixture was then stored at 20°C. After the PCR apparatus reached room temperature, 10μL of PCR reaction solution was added to each tube and incubated in the thermocycler for 35 cycles at 95°C for 30 s, 60°C for 30 s, and 72°C for 60 s. Following 35 cycles, DNA was incubated at 72°C for 20 min and stored at 15°C. The PCR products were subjected to capillary electrophoresis on ABI 3500xL Genetic Analyzer, and the resulting data were analyzed using Coffalyser software (Coffalyser.NET.v1.0). The relative copy number was calculated according to the Product Description of SALSA® MLPA® Probemixes.

**4. Custom-designed liquid phase chip**

This method was primarily employed to determine the CNV found in the 16p13.3 region associated with thalassemia. The kit was developed by our laboratory based on the barcode magnetic bead technology (http://www.apbiocode.com/) of Applied BioCode company in the United States. The specific experimental steps and required instruments can be referred to in the literature [2]. The main steps included PCR amplification, hybridization, and chip detection (performed at MAGPIX workstation, Luminex xMAP Technology).

**5. qPCR**

According to the range indicated by CES detection, one pair of specific qPCR primers was designed within each target CNV, with the *RPP30* gene (coding Ribonuclease P/MRP, 30-KD subunit) serving as the internal reference. The reagents (supplied by Accurate Biology) comprised SYBR Green *Pro Taq* HS mix (10 μL), forward and reverse primers (0.6 μL each), ROX dye (0.2 μL), DNA template (1 μL), plus ddH_2_O to a total volume of 20μL. The amplification reaction was conducted on the Applied Biosystems 7500 Real-Time PCR System with pre-denaturation at 95℃ for 2 min, followed by 45 cycles of denaturation at 95℃ for 15 s, annealing at 56℃ for 20 s, and extension at 65℃ for 40 s. The copy number of the target region was determined through the relative quantitative calculation of Ct values.

**6. Gap-PCR**

Multiple pairs of PCR primer were designed for amplification around the verified CNV. The long-range PCR reaction system consisted of 12.5 μL of LA-Taq premix (TaKaRa LA Taq), 1.5 μL of each forward and reverse primer at a concentration of 10 μM, 7.5 μL of 5 M of betaine, and 2 μL of DNA template. The reaction procedure included pre-denaturation at 95℃ for 5 min, denaturation at 95℃ for 30 s, annealing at 61℃ for 10 min (38 cycles), extension at 61℃ for 7 min, followed by storage at 25℃. The amplified fragment was found to be smaller than expected, possibly containing the breakpoint. Subsequently, Sanger sequencing was performed on this product to confirm the exact CNV size. The obtained sequences were compared with those retrieved from the UCSC Genome Browser (http://genome.ucsc.edu).

**Reference**

1. Yin, A.H., et al., *Noninvasive detection of fetal subchromosomal abnormalities by semiconductor sequencing of maternal plasma DNA.* Proc Natl Acad Sci U S A, 2015. 112(47): p. 14670-5.

2. Yin, A., et al., *The prevalence and molecular spectrum of alpha- and beta-globin gene mutations in 14,332 families of Guangdong Province, China.* PLoS One, 2014. **9**(2): p. e89855.
